# Actin Binding to the BAR Domain and Arf GAP Activity of ASAP1 Coordinately Control Actin Stress Fibers and Focal Adhesions

**DOI:** 10.1111/boc.70005

**Published:** 2025-04-07

**Authors:** Hye‐Young Yoon, Jonah Unthank, Sandeep Pallikkuth, Pei‐Wen Chen, Paul A. Randazzo

**Affiliations:** ^1^ Center for Cancer Research, National Cancer Institute Bethesda Maryland USA; ^2^ Department of Biology Williams College Williamstown Massachusetts USA

## Abstract

**Background:**

Actin stress fibers (SFs) and focal adhesions (FAs) are dynamic structures crucial to a range of cell behaviors including cell morphology, cell migration, proliferation, survival, and differentiation. The Arf GAP ASAP1 affects both SFs and FAs. Here, we test the hypothesis that two domains with distinct biochemical activities in ASAP1, the BAR domain that binds actin and nonmuscle myosin 2 (NM2) and the Arf GAP domain, which is necessary for inducing hydrolysis of GTP bound to Arf, coordinately regulate the structures.

**Results:**

We found that ASAP1 associated with bundled actin, including SFs, colocalizing with α‐actinin and nonmuscle myosin 2A (NM2A), and with paxillin in FAs. Reducing ASAP1 expression altered both SFs and FAs in four cell lines that we examined. The effects of reducing ASAP1 expression could be reversed by ectopic expression of ASAP1. Reduced expression of Arf5, a substrate for ASAP1, or expression of either dominant negative or GTPase deficient mutants of Arf5, affected SFs and FAs similarly to ASAP1 knockdown. Both an active GAP domain and a BAR domain contained in the same ASAP1 polypeptide were necessary to maintain FAs and SFs.

**Conclusions and Significance:**

Taken together, the results support the idea that ASAP1 coordinates the maintenance of FAs and SFs through integrated function of the BAR and GAP domains. We speculate that ASAP1 regulates SFs and their interaction with FAs through direct binding to components of the actin cytoskeleton. We discuss hypotheses related to this Arf‐dependent activity of ASAP1 and propose the function of ASAP1 is not control of Arf•GTP levels.

## Graphical abstract

Dynamic assembly of higher order actin cytoskeletal structures is fundamental to metazoan biology. While the regulation of actin filaments and the constituents of higher order actin structures have been described, the mechanism underlying the regulated assembly of the higher order actin structures, such as actin stress fibers, is still being discovered. Here, we found that a cycle of GTP binding and hydrolysis by the GTPase Arf is linked to the control of stress fibers through the Arf GTPase‐activating protein ASAP1. We propose that ASAP1 is a machine linking two precursors of actin stress fibers to form the fibers which depends on Arf‐GTP binding to ASAP1 and subsequent hydrolysis of the bound GTP. Thus, ASAP1 might function as a noncanonical effector of Arf to control stress fiber assembly.

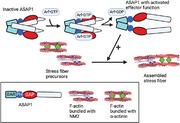

## 1 Introduction

Actin stress fibers (SFs) and focal adhesions (FAs) are integrated structures important for cell contraction, adhesion and signaling, and mediate cell behaviors including migration, proliferation, survival, and differentiation (Carragher and Frame 2004; Gardel et al. 2010; Geiger et al. 2009; Hehlgans et al. 2007; Schoenwaelder and Burridge 1999; Tojkander et al. 2012; Wehrle‐Haller and Imhof 2002; Wozniak et al. 2004). The formation of FAs depends on intracellular trafficking and activation of integrins and incorporation of integrins with structural and signaling proteins into a plaque. The maturation of FAs has been reported to depend on SFs (Livne and Geiger 2016). There has been recent progress in understanding the assembly of SFs and the relationship to FAs. There are four types of SFs, including transverse arcs, dorsal SFs, ventral SFs, and nuclear caps (Tojkander et al. 2012). The structures might be independently regulated. Transverse arcs are not connected to FAs (Tojkander et al. 2012), and FAs can be formed when SF formation is prevented or altered by either knockdown of tropomyosin (Gateva et al. 2017; Kumari et al. 2024) or knockdown of ASAP3 (Ha et al. 2008). The mechanisms coordinating SFs and FAs are still being discovered. The Arf GTPase pathway might be central to control of these structures. One component of the Arf pathway, the Arf GTPase‐activating protein, ASAP1, affects both FAs and SFs (Chen et al. 2020; Chen et al. 2016; Gasilina et al. 2019; Gasilina et al. 2022; Randazzo et al. 2000).

ASAP1 is a 130 kDa protein composed of BAR, PH, ArfGAP, Ankyrin repeat, proline rich, E/DLPPKP repeat, and SH3 domains (Brown et al. 1998). It homodimerizes through the BAR domain (Jian et al. 2009; Nie et al. 2006). First discovered as an Arf GAP and as an Src‐binding protein (Brown et al. 1998), ASAP1 was found to associate with and regulate FAs, with the regulation of cell spreading dependent on GAP activity (Randazzo et al. 2000). More recently, ASAP1 was discovered to bind to nonmuscle myosin 2 (NM2) (Chen et al. 2016) and F‐actin (Chen et al. 2020; Gasilina et al. 2019; Gasilina et al. 2022) via the BAR domain, and to be necessary for SF formation (Gasilina et al. 2022). ASAP1 mutants deficient in F‐actin binding did not support SF formation (Gasilina et al. 2022). The effect of the BAR domain on FA formation and the effect of the GAP activity on SFs and FAs have not been examined. A plausible hypothesis is that the two functions of ASAP1 cooperate to coordinate SF formation and FA maturation.

Here, using mutants of Arf and ASAP1, we examine the effects of actin binding and Arf GAP activity of ASAP1 on FAs and SFs. ASAP1 was found to localize to both FAs and SFs. Cells with reduced ASAP1 expression had reduced numbers and size of FAs and reduced or altered SFs. Ectopic expression of ASAP1 but not ASAP1 mutants lacking the BAR domain or having reduced GAP activity rescued the effect of reduced expression of endogenous ASAP1 on FAs and SFs. The GAP activity is unlikely acting independently of actin binding as expression of Arfs that bound GTP poorly (often referred to as dominant negative or DN) had a similar effect on SFs and FAs as ASAP1 knockdown. Coexpression of GAP‐dead ASAP1 and ASAP1 lacking the BAR domain in trans did not rescue the phenotype of ASAP1 knockdown. Based on these results, we propose that actin binding and GAP activity are integrated to coordinately regulate FAs and SFs.

## 2 Materials and Methods

### 2.1 Cell Culture and Antibodies

Human U2OS cells and cell lines derived from U2OS were maintained in McCoy's 5a medium supplemented with 10% heat inactivated fetal calf serum (FCS). Human HeLa cells, and mouse cells (NIH3T3) fibroblast and C2C12 (myoblast) were cultured in high glucose Dulbecco's modified Eagle's medium (DMEM) containing 4 mM l‐glutamine, 1 mM sodium pyruvate, and 10% FCS. All cultured media contained 100 U/mL penicillin and 100 µg/mL streptomycin (ThermoFisher Scientific), and all cells were cultured in a humidified incubator of 5% CO_2_ at 37°C. For passaging, cells were detached with 0.05% trypsin‐EDTA and subsequently replated.

VectorBuilder (Chicago, IL) generated the stable cell lines expressing ASAP1a‐HA, [ΔBAR]ASAP1a‐HA (with residues 1–325 of ASAP1a deleted) and [R485K]ASAP1a‐HA under control of a tetracycline promoter. Briefly, synthesized gene inserts of wild type (WT) and mutant HA‐tagged ASAP1 were cloned into a tetracycline‐inducible lentiviral backbone using Gateway cloning, verified by sequencing and transfected into packaging cells for production of lentiviral particles. U2OS cells were transduced with resulting lentivirus for generation of stable cell lines. Cell lines expressing [ΔBAR]ASAP1a‐HA and [R485K]ASAP1a‐myc under control of a tetracycline promoter were generated by transducing [ΔBAR]ASAP1a‐HA expressing cell lines with lentivirus containing the tetracycline‐inducible lentivirus backbone with insertion for the ORF for [R485K]ASAP1a‐myc. For rescue experiments, ASAP1 was knocked down with dicer substrate RNA duplex targeting the 3′UTR (Integrated DNA Technologies [IDTs]) in stable U2OS cell lines using Dharmafect 1 (Horizon Discovery). Expression of empty vector, WT or mutant ASAP1 was induced by addition of doxycycline 48 h postdicer transfection.

Primary Abs used for these studies included: rabbit (Rb) anti‐Arf5 (ARF1960) from ThermoFisher Scientific. Mouse (Ms) anti‐HA (6E2), Ms anti‐paxillin (Clone 349), Ms anti‐GAPDH (14C10), Ms anti‐α‐Actinin, and Rb anti‐Arf6 (D12G6), Rb anti‐HA (C29F4), Rb anti‐Myosin IIa (E7Y9O), and Rb anti‐β‐Actin Abs from Cell Signaling. Ms anti‐Arf1 (AT1B3) and Ms anti‐DDEF1 (aka ASAP1) Abs (2G7) were from Abnova. Rabbit anti‐ASAP1 antisera (antisera 642) was raised to C‐terminal antigens developed in our lab (Randazzo et al. 2000).

For immunofluorescence, donkey anti‐Ms or Rb secondary Abs conjugated to Alexa Fluor488, 568, or 647 were sourced from ThermoFisher Scientific. For actin staining, AlexaFluor488‐, 568‐, or 594‐phalloidin were obtained from Invitrogen. NucBlue Fixed Cell ReadyProbes reagent (DAPI) was used for nuclear counterstain in the cells (ThermoFisher Scientific). Secondary Abs for immunoblotting, IRDye680 donkey anti‐mouse IgG and IRDye800 donkey anti‐rabbit IgG, were from LiCor.

Expression vectors for Arf1‐His_6_‐HA, [T31N]Arf1‐His_6_‐HA, [I46D]Arf1‐His_6_‐HA, Arf5‐His_6_‐HA, [T31N]Arf5‐His_6_‐HA, and [I46D]Arf5‐His_6_‐HA under a CMV promoter were synthesized by Genscript. The histidine tandems reduce the effect of the negatively charged HA tag on function of Arf.

### 2.2 Transfections

Plasmid transfections were performed using FuGENE HD transfection reagent from Promega following the manufacturer's instructions. Briefly, 2.7 × 10^5^ cells were plated on a 6‐well plate 24 h before transfection. Two microgram of plasmid DNA was mixed with 7 µL of transfection reagent (1:3.5 ratio) in 100 µL of OPTi‐MEM media. The mixture was incubated for 5–10 min at room temperature (RT) before adding it to cells in media containing 10% FCS. After transfection, cells were incubated for 24 h before downstream experiments.

siRNA transfection was performed using DharmaFECT 1 transfection reagent (Horizon Discovery) as per the manufacturer's instructions for either siRNA or dicer substrate RNA duplex (DsiRNA).

For rescue experiments, U2OS cell lines expressing the indicated proteins under control of the tetracycline promoter were first transfected with 10 nM DsiRNA targeting the 3′‐UTR of human ASAP1 or a nontargeting DsiRNA (IDT) using the DharmaFECT1 transfection reagent. Twenty‐four hours after transfection, the cells were treated with 0.5 µg/mL doxycycline to induce the expression of the empty vector, WT, or mutant ASAP1, and then incubated for an additional 48 h.

The following siRNA were used: DsiRNA against human Arf1 (#1; 5′ – CAAUUCUGCAUGGUCACAGUAGAGA – 3′, #2; 5′ – AAGAAUCCAAGUCGAGAACACUUGA – 3′, or #3; 5′ – AAACCGUGGAGUACAAGAACAUCAG – 3′), human ASAP1 (#2; 5′ – AUGCAUUUAGAAGCUUACCUGAAAT – 3′), and negative control DsiRNA (called control SiRNA in text), all purchased from IDTs. We obtained siGenome siRNA against human Arf5 (#17; 5′ – GCUUGGAUGCGGCUGGCAA – 3′, SMARTpool, which includes four pooled siRNAs; 5′ – CCACAAUCCUGUACAAACU – 3′, 5′ – UGAGCGAGCUGACUGACAA – 3′, 5′ – CUGAUGAACUCCAGAAGAU – 3′, and 5′ – GCUUGGAUGCGGCUGGCAA – 3′) and siGENOME nontargeting siRNA #5 (5′ – UGGUUUACAUGUCGACUAA – 3′, (called control SiRNA in text) as a control from Horizon Discovery.

### 2.3 Immunofluorescence Microscopy

Cells were plated on fibronectin (10 µg/mL)‐coated coverslips for 5.5 h in OPTi‐MEM medium. After fixation in 4% paraformaldehyde in PBS for 15 min, the cells were washed three times with PBS, then permeabilized in blocking solution (1% bovine serum albumin [BSA], 2.5% FCS and 0.2% saponin in PBS) for 20 min. The cells were Incubated with primary and fluorescently labeled secondary Abs and/or phalloidin for 1 h at RT in blocking solution. Next, the cells were incubated with a DAPI mixture (two drops of DAPI solution in 1 mL PBS) for 5 min at RT in the dark for nuclear counterstaining. After three 5‐min washes with PBS, coverslips were mounted with DAKO mounting medium.

For the detection of nonmuscle myosin IIa using Rb anti‐Myosin IIa Ab labeled with FlexAble CoraLite Plus 555 Ab labeling kit from Proteintech, all reagents were warmed to RT before use. 0.5 µg of the primary Ab was mixed with 1 µL of FlexLinker, and added FlexBuffer to a total volume to 8 µL. The mixture was gently mixed and incubated for 5 min at RT in the dark, followed by the addition of 2 µL of FlexQuencher and another 5 min incubation in the dark. The labeled Ab was then diluted 1:50 in blocking solution.

Confocal imaging was conducted using a Leica TS SP8 confocal laser scanning microscopy with optimized z‐stack parameters. The set up included a pinhole of 1.00 Airy Unit, an HC PL APO CS 63×/1.4 oil objective, a scan speed of 700 Hz, XY = 1024 × 1024, and a zoom factor of 1.78. For a colocalization studies, lightning imaging was performed with the following settings: refractive index 1.2, custom mounting medium, an HC PL APO CS 63×/1.4 oil objective at 700 Hz, XY = 3696 × 3696, and a zoom factor of 1.28, including a z‐stack. When a wider view of the cells was needed, the fixed cells were captured using a tile scan.

For each experiment, a series of test images were captured to determine exposure settings that minimized oversaturation; this setting was then applied to all conditions. 16‐bit images were analyzed using Leica LAS X software.

### 2.4 Image Analysis

Bundled actin, which includes SFs, was quantified by two methods that yielded similar results. In one approach, bundles of phalloidin stained actin were detected using the Ridge detection plugin from ImageJ with parameters: line width 5, high contrast 250, low contrast 10, sigma1.75, low threshold 0.0, upper threshold 3.5, min line width 10, and max line width 0. In a second approach, two ∼80 pixels x 80 pixel ROIs were drawn on each cell in a z‐stack projection. Two orthogonal sets of nine parallel lines, each line separated by 5 pixels were drawn through the center of the ROIs. The number of peaks in line profiles was determined. The set of parallel lines yielding the maximum number of peaks was used as a measure of bundled actin within each ROI, which are the data presented in the figures.

Paxillin staining was used as a marker of FAs. FA number and size were analyzed as previously described (Chen and Kroog 2010). Cellular distribution of FAs was analyzed by plotting the density of FAs as a function of the relative distances from the edge of the cell, 0 being the edge of the cell.

### 2.5 Western Blotting

To assess the knockdown or overexpression of the interested proteins, cell lysate in 2X Laemmli sample buffer (Bio‐Rad) with 0.05% β‐mercaptoethanol and Halt protease and phosphatase inhibitor cocktail (ThermoFisher Scientific) was resolved on 4%–20% Criterion TGX precast midi protein gel (Bio‐Rad), then transferred to nitrocellulose membrane (Bio‐Rad). After blocking for 1 h at RT in Intercept TBS blocking buffer (LI‐COR), the membranes were incubated overnight at 4°C with primary Abs diluted in intercept TBS Ab diluent (LI‐COR). Following three 5‐min washes with TBS containing 0.1% Tween‐20 (TBST) at RT, the membranes were incubated with IRDye‐labeled secondary Abs (LI‐COR) for 1 h in Intercept TBS blocking buffer with 0.1% Tween‐20 in the dark, washed thrice in TBST, once in TBS, and then scanned using a LI‐COR Odyssey DLx imaging system. Scanned band intensities were quantified using Image Studio Lite (LI‐COR).

### 2.6 Statistical Analysis

Statistical analyses were carried out using Prism 8 (GraphPad). Comparison of two groups was carried out using Student's *t* test, and comparison of data sets with more than two groups was carried out using ANOVA. Alpha was set to 0.05 for all experiments. Values represented are mean ± s.e.m., unless otherwise noted.

## 3 Results

### 3.1 ASAP1 Colocalizes With FAs and F‐Actin, α‐Actinin, and Nonmuscle Myosin 2A (NM2A)

ASAP1 localizes to several structures, including circular dorsal ruffles, invadopodia (Bharti et al. 2007; Chen et al. 2016; Randazzo et al. 2000; Randazzo, Inoue, and Bharti 2007) and, in resting cells, FAs (Randazzo et al. 2000); however, localization of ASAP1 relative to other actin structures and actin‐associated proteins in resting cells has not been examined. As a first step to assess the potential roles of ASAP1 in the control of FAs and the related structures, SFs, the localization of ASAP1 relative to actin, paxillin, α‐actinin, and NM2A in cells spread on fibronectin was determined using the Lightning module for a Leica confocal microscope. Two mouse cell lines, NIH 3T3 fibroblasts and C2C12 myoblasts, and two human cell lines, U2OS, derived from an osteosarcoma, and HeLa cells, were examined. We found ASAP1 colocalized with paxillin in FAs, extending into the distal tips of ventral SFs (Figure 1A, B, Figure S1A, B). At the proximal ends of FAs, ASAP1 also colocalized with α‐actinin and NM2A (Figure 1A, B, Figure S1A, B). ASAP1 localization was not restricted to FAs, which was most pronounced in NIH 3T3 fibroblasts and C2C12 myoblasts (Figure 1A and Figure S1A). ASAP1 was in periodically distributed puncta with possible partial overlap with puncta containing α‐actinin and NM2A (Figure 1A, B, Figures S1A, B, examples indicated with arrowheads in all figures) including regions devoid of paxillin, and in SFs (Figure 1A, lower panels, area indicated with arrowheads). Pearson's coefficients indicated that ASAP1 might partly colocalize with paxillin in all cells and with α‐actinin and NM2A (Figure 1C), with the greatest level of possible colocalization of ASAP1 with α‐actinin and NM2A observed in NIH 3T3 and C2C12 (The differences in the extent of ASAP1 localization with α‐actinin and NM2A between human and mouse cells might result from antibody (Ab) recognition: while the Ab we used is the best we were able to find for staining for both mouse and human ASAP1, it recognizes the mouse protein better than human).

FIGURE 1Localization of ASAP1 relative to cytoskeleton components. (A) C2C12, (B) U2OS. The indicated cells were plated on fibronectin coated coverslips, immunostained for ASAP1 and actin, and either nonmuscle myosin 2A (NM2A), α‐actinin or paxillin and imaged using a Leica TS SP8 confocal laser scanning microscopy with a lightening module. Boxed regions in top panels are enlarged in the lower panels with arrowheads indicating colocalization of ASAP1 with α‐actinin (i) and nonmuscle myosin 2 (NM2) (ii), and areas where ASAP1 does not colocalize with paxillin (iii). Note that at the edge of the cells, ASAP1 and paxillin extensively colocalized as indicated by the Pearson's coefficient presented in (C) and described previously. (C) Colocalization of ASAP1 with NM2A, α‐actinin, and paxillin. Pearson's coefficients were determined for the indicated pairs of proteins in the indicated cell lines. Scale bars in (A) are 50 µm. In (B), scale barfs are 25 µm.

**Figure:**
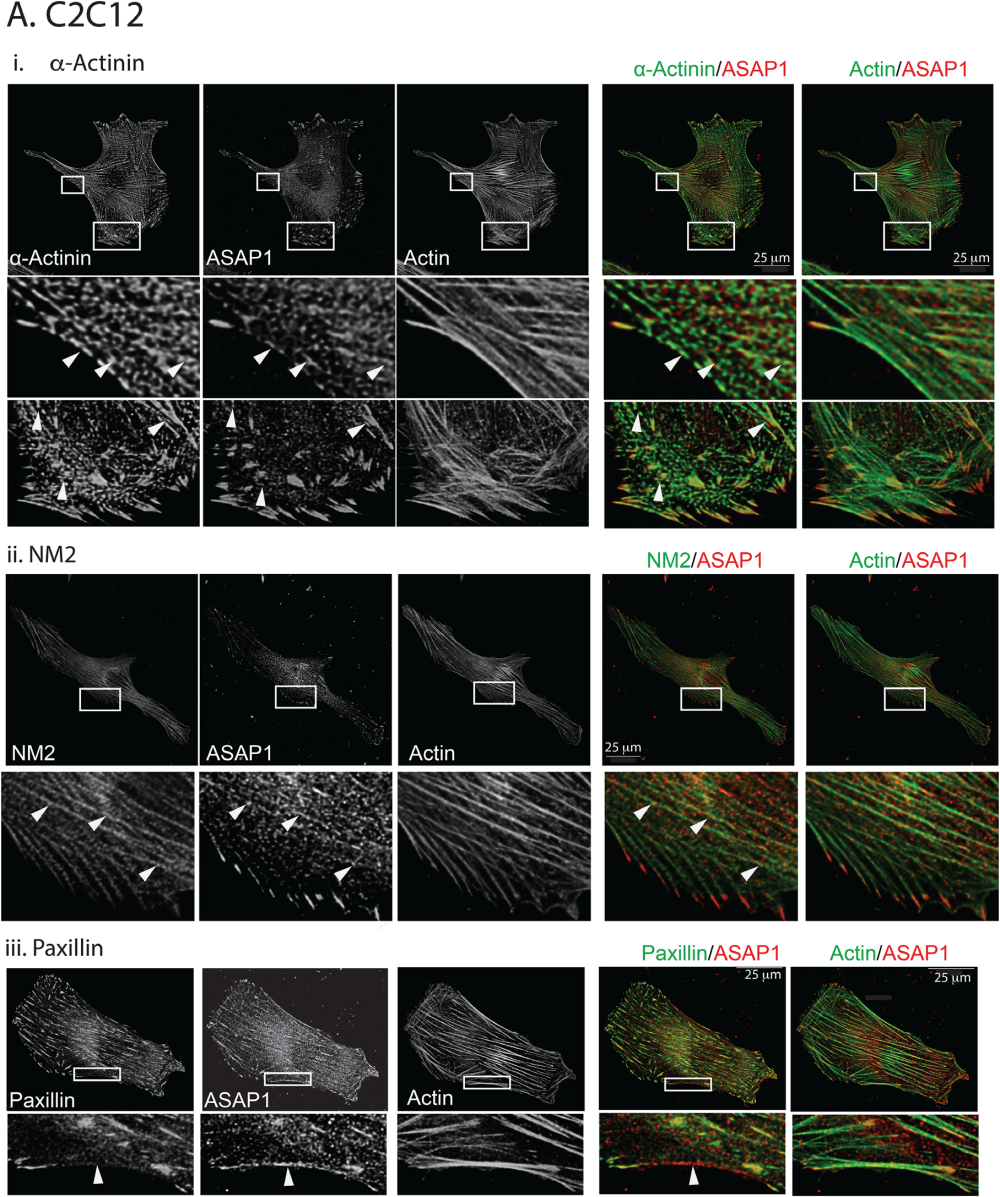

**Figure:**
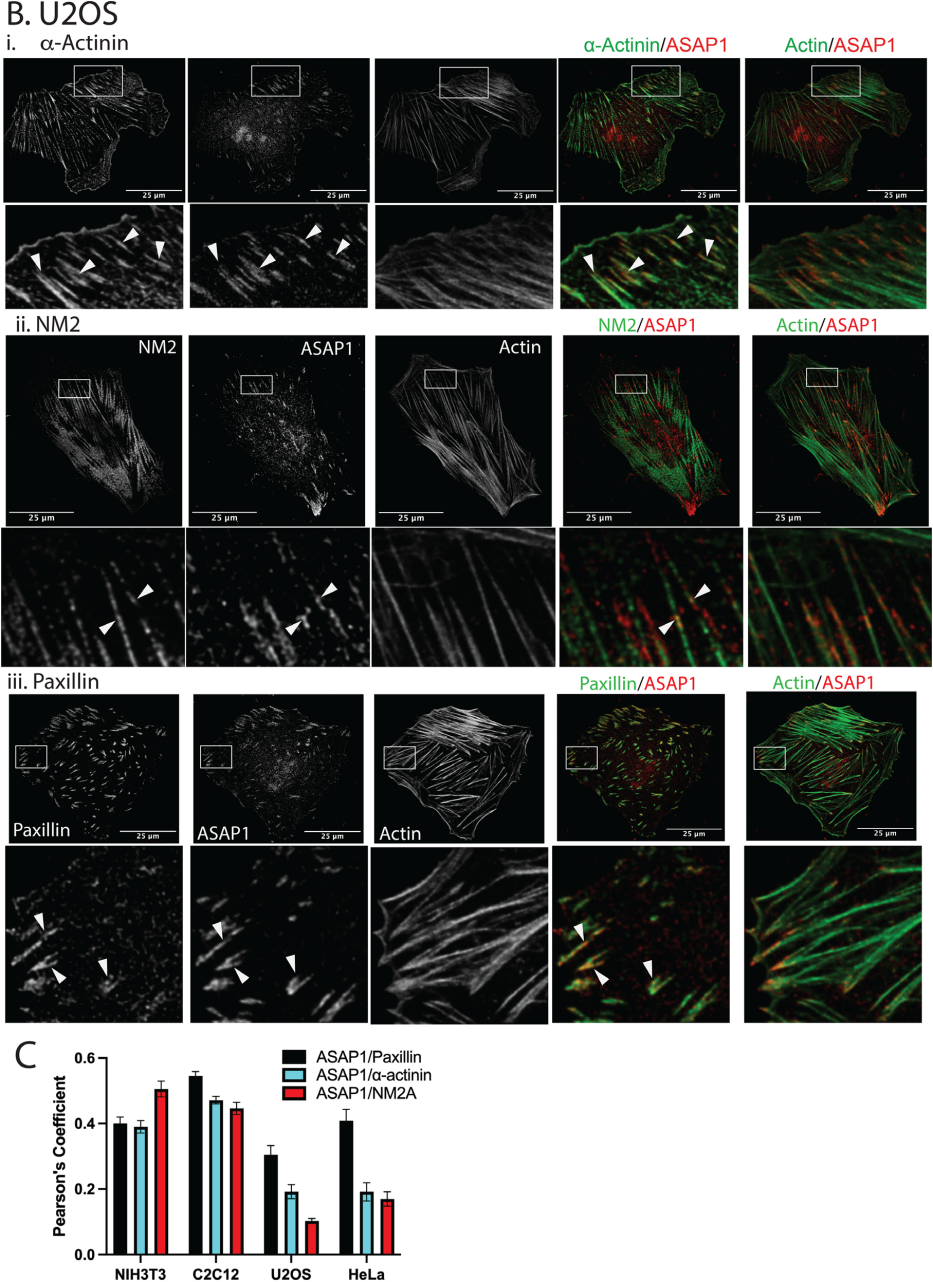

### 3.2 Reduced ASAP1 Expression Affects Both FAs and SFs

**FIGURE 2:**
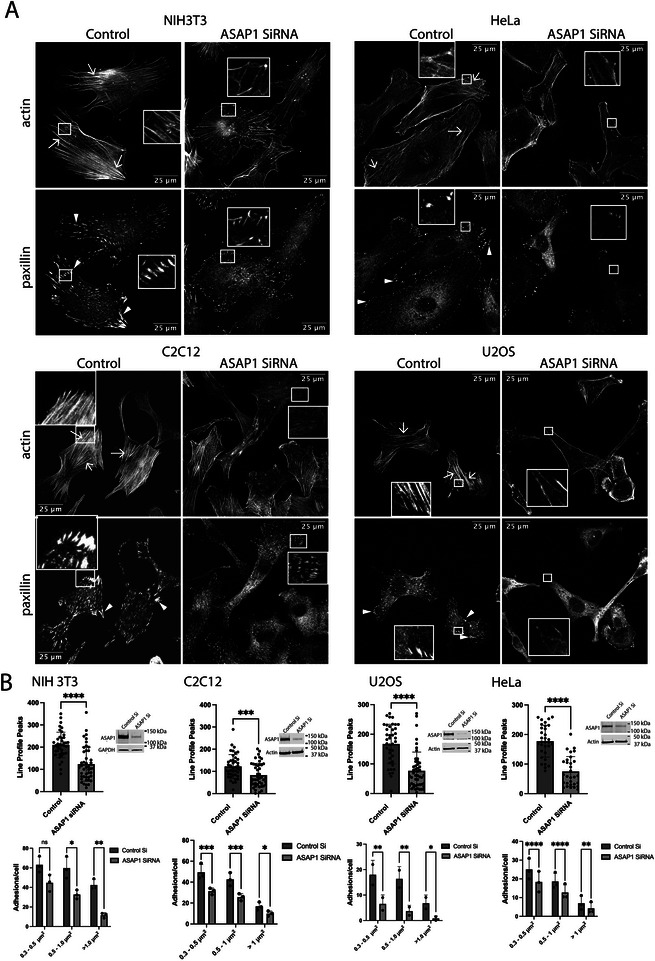
Changes in both focal adhesions (FAs) and stress fibers (SFs) are common to four cell lines treated with siRNA targeting ASAP1. The indicated cells were treated with either nontargeting (control) or ASAP1‐targeting siRNA for 3 days. The cells were harvested, ASAP1 levels determined (Panel B) and plated on fibronectin‐coated cover slips for 5–6 h. The cells were then fixed and prepared for immunofluorescence microscopy staining for actin and paxillin. (A) Representative images. Arrows point to examples of SFs and arrowheads point to examples of FAs. (B) Quantifications of bundled actin and FAs. Bundled actin was determined as described in “Section 2.” Briefly, profiles of two orthogonal sets of nine lines in two ∼80 pixel boxes/cell for 10–20 cells were determined. The number of peaks for each set of orthogonal lines was determined and the maximum for a region of interest is reported. Results from one of two experiments with similar results are presented. Cellular FAs were quantified as previously described (Chen and Kroog 2010). The summary of three experiments for C2C12, NIH3T3, and HeLa cells and of two experiments for U2OS are shown. The inset is an immunoblot for ASAP1 in cells treated with control or ASAP1 targeted siRNA. *t* tests were used for analysis of bundled actin and two‐way analysis of variance (ANOVA) for FAs. *p < 0.05; **p < 0.01; ***p < 0.001; ****p < 0.0001.

**FIGURE 3:**
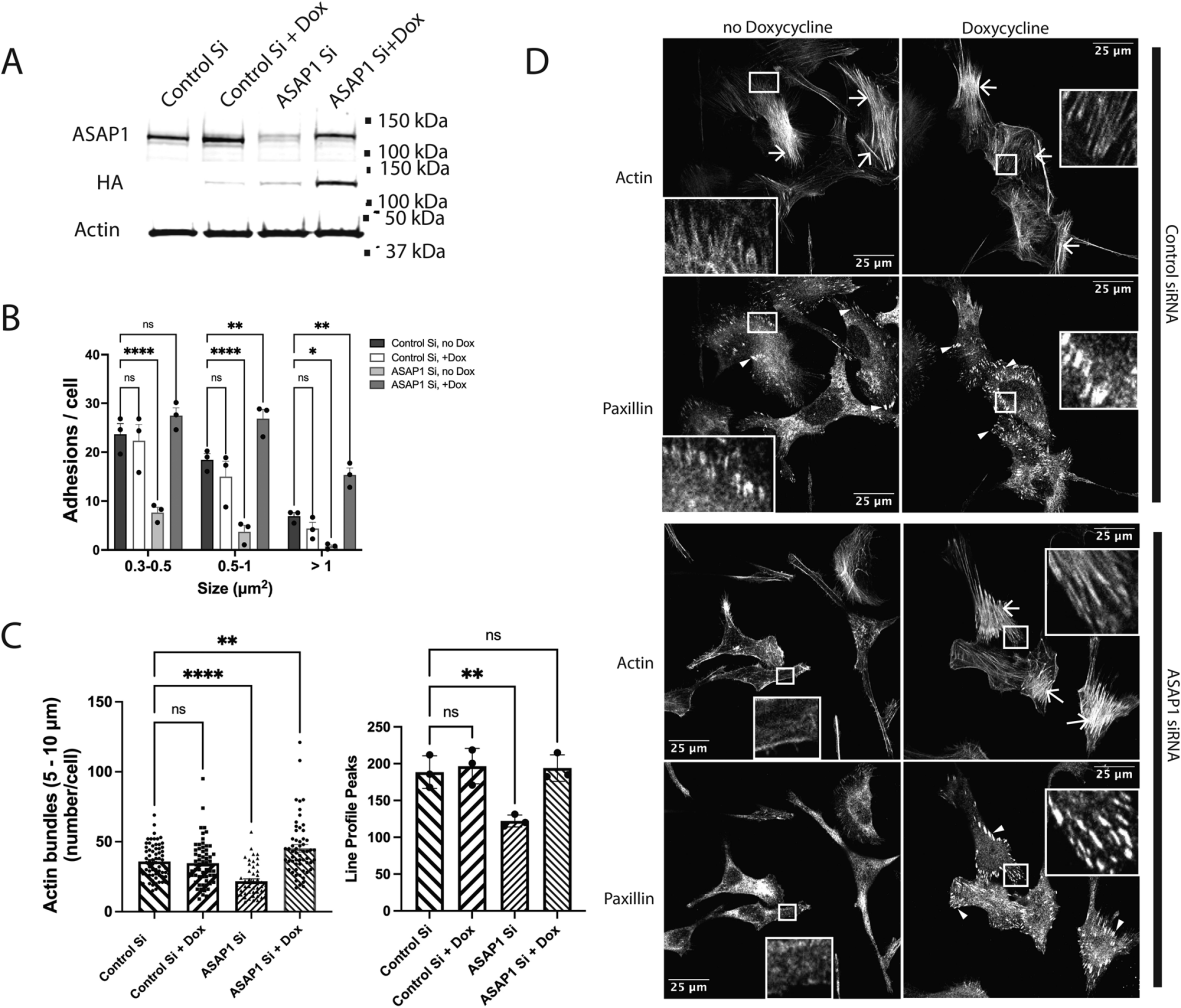
Effect of knockdown of endogenous ASAP1 on focal adhesions (FAs) and stress fibers (SFs) is rescued by expression of ASAP1 from an ectopic promoter. U2OS cells, with cDNA expression cassettes for ASAP1 under control of a tetracycline promoter integrated into the chromosome using a lentivirus vector, were plated in 35 mm cell culture plates and treated with either nontargeting (control Si) or siRNA targeting the 3′UTR of ASAP1 (ASAP1 siRNA) as indicated. After 24 h, doxycycline (0.5 µg/mL) was added as indicated. Two days later, cells were harvested and replated on fibronectin coated cover slips for 6 h in serum free medium. The cells were then fixed and stained for actin and paxillin. (A) Immunoblot of cell lysates for ASAP1 under the indicated conditions. The top panel is a blot using an antibody to ASAP1, the middle panel uses an antibody to the epitope tag fused to the ectopically expressed ASAP1. Note there is some expression without doxycycline when endogenous ASAP1 is knockdown and expression with doxycycline is greater with reduced endogenous ASAP1. This observation is reproducible and is the subject of another project. β‐Actin was used as a loading control. (B) Quantification of FAs. Conditions are indicated in the figure. (C) Quantification of bundled actin. Bundled actin was quantified using the imageJ plugin RidgeDetector (left panel) and by determining the number of peaks in nine line profiles as described in Figure 2 and “Section 2” (right panel). Results using either method for quantification were similar. (D) Representative images. SFs are indicated with arrows and FAs with arrowheads. Results were analyzed with one‐way (Panel C) and two‐way (Panel B) analysis of variance (ANOVA). *p < 0.05; **p < 0.01; ***p < 0.001; ****p < 0.0001.

As an initial test of the hypothesis that ASAP1 coordinately regulates FAs and SFs, we determined the effect of reducing ASAP1 expression on FAs and SFs in the four cell lines used to examine ASAP1 localization. siRNA efficiently reduced ASAP1 expression in all cells we examined (Figure 2B). Reduced ASAP1 resulted in a reduction in the number and size of FAs (indicated by arrowheads) and SFs (arrows). SFs that formed were thinner and often disorganized (Figure 2A) (Two methods described in Section 2 were used to quantify bundled actin, taken as a measure of SFs, with similar results). Off‐target effects were excluded using U2OS osteosarcoma cells expressing ASAP1 under a tet‐on promoter (Figure 3). As in the parental cells, there was a reduction of FAs and SFs by reducing ASAP1 expression with an siRNA targeting the 3′UTR of the message. The effect of reducing ASAP1 by siRNA was reversed by inducing ASAP1 expression with doxycycline. In some experiments, the increased expression of ASAP1 from the tet‐on promoter led to greater numbers and bigger FAs, and more bundled actin.

### 3.3 Reducing Arf5 Expression Partly Recapitulates the Effect of ASAP1 Knockdown on SFs and FAs

**FIGURE 4:**
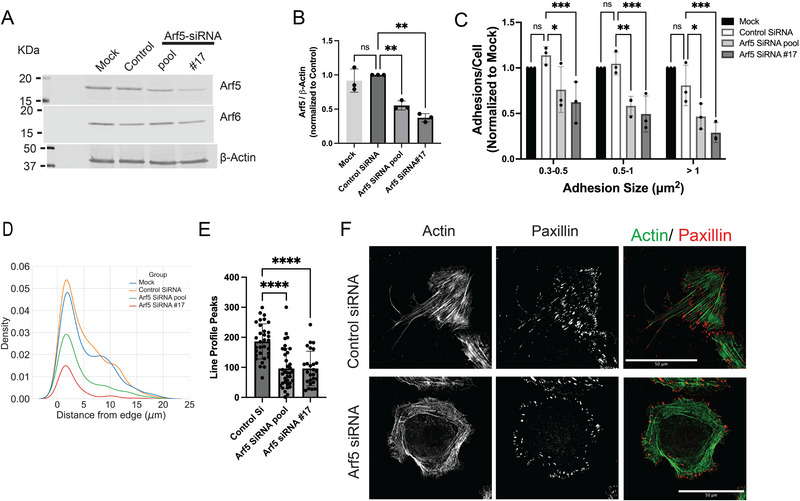
Reduced Arf5 expression affects stress fibers (SFs) and focal adhesions (FAs). U2OS cells were treated with either a pool of siRNA or a single siRNA targeting Arf5. After 3 days, cells were harvested. Aliquots were lysed and immunoblotted. Cells were plated on fibronectin coated coverslips for 5–6 h, fixed, stained for actin and paxillin, and imaged. (A) Immunoblot of lysates of U2OS cells treated with the indicated siRNA for the reduction of Arf5 expression. Specificity of knockdown was checked by blotting for Arf6. β‐Actin was used as a loading control. (B) Quantification of the Immunoblot. Blots were quantified using fluorescent secondary antibodies detected using an Odyssey Imager (LiCOR). (C) Effect of Arf5 knockdown on FAs in U2OS. FAs were quantified as described in Figure 2 and in “Section 2.” (D) Effect of Arf5 knockdown on cellular distribution of FAs. The distance of FAs (>1 µm^2^) from the cell edge was determined as described in “Section 2.” The density of paxillin containing plaques at relative distances from the edge of the cell is plotted, 0 being the edge of the cell. Thirty‐eight cells for the mock condition, 40 cells for the control siRNA condition and 31 cells each for the Arf5 targeted siRNA condition were analyzed. (E) Effect of Arf5 knockdown on SFs. Bundled actin was quantified as described in Figure 2 and in “Section 2.” (F) Representative images. Cells were treated with nontargeting (control) or Arf5 targeted (#17 supplied by Dharmacon) siRNA. For Panel (C), summary of three experiments is presented. Each experiment was normalized to the mock transfection control. The data was analyzed using two‐way analysis of variance (ANOVA) with a repeated measures design. Panels (B) and (E) were analyzed using one‐way ANOVA. *p < 0.05; **p < 0.01; ***p < 0.001; ****p < 0.0001.

We examined the effect of perturbing Arf1 and Arf5, which are ASAP1 substrates in vitro, on SFs and FAs in U2OS osteosarcoma cells. Arf1 expression was reduced by greater than 90% using three siRNAs (Figure S2A). One of the three siRNAs reduced the number of FAs between 0.5 and 1 µm^2^ (Figure S2B); however, given the other siRNAs reduced Arf1 to the same extent but had no effect on FAs, the reduction of FAs is likely an off‐target effect. None of the siRNA targeting Arf1 affected SFs (Figure S2C). In contrast, reduction in Arf5 expression affected both FAs and SFs. Arf5 expression was reduced with a single siRNA and a pool of siRNA (Figure 4A). Maximum reduction was about 70% with the single siRNA and ∼50% with the pool (Figure 4B). Cells with reduced Arf5 levels had fewer FAs (Figure 4C), which were restricted to the cell edge (Figure 4D) and less bundled actin (Figure 4E). Representative images are shown in Figure 4F.

### 3.4 Both GTP Binding and Hydrolysis by Arf5 Are Necessary for Maintenance of SFs and FAs

To complement the knockdown experiments, we examined the effect of expressing Arf1‐HA and Arf5‐HA, [T31N]Arf1‐HA and [T31N]Arf5‐HA (low affinity for GTP, hence can be considered GDP‐locked), and [I46D]Arf1‐HA and [I46D]Arf5‐HA (binds to ASAP type Arf GAPs with similar affinity as WT Arf but selectively resistant to ASAP‐induced GTP hydrolysis and, therefore, can be considered GTP‐locked, constitutively active by prevailing paradigms [Luo et al. 2005]) in U2OS cells. The advantage of using the mutants is that the knockdown experiments might be confounded by redundancy. The disadvantage is that the mutants might not be specific for Arf1 or Arf5 targets. The distributions of WT Arf1‐HA and [I46D]Arf1‐HA were primarily perinuclear (Figure S3A). [T31N]Arf1‐HA was distributed diffusely throughout the cell (Figure S3A). Expression of [T31N]Arf1‐HA reduced FAs and changed the distribution of the FAs in cells, with a greater fraction of the FAs localized to the cell periphery (Figure S3A–C). [I46D]Arf1‐HA did not significantly change the number of FAs (Figure S3A–C). Ectopic expression of either Arf1‐HA, [T31N]Arf1‐HA, or [I46D]Arf1‐HA reduced the number of SFs compared to the empty vector control (Figure S3A, D).

**FIGURE 5:**
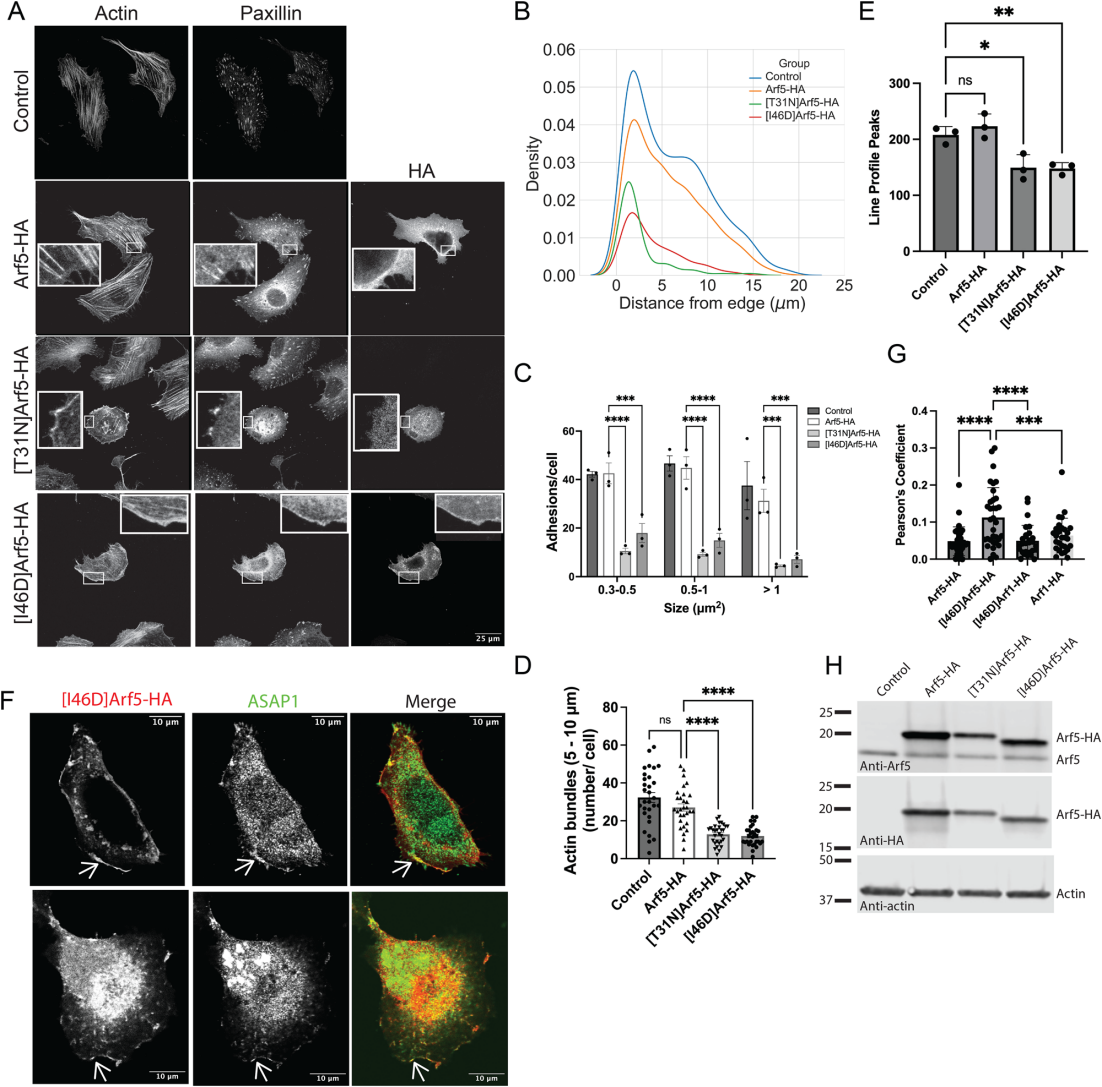
Both GTP binding and hydrolysis by Arf5 are required for the maintenance of focal adhesions (FAs) and stress fibers (SFs). (A) Representative images of U2OS cells expressing the indicated mutant of Arf5. Cells were transfected with plasmids for expression of the indicated Arf5 mutant. Control cells were treated with transfection reagent and an empty expression vector. After 24 h, the cells were replated on fibronectin coated coverslips for 6 h, fixed and stained for actin, paxillin, or the ectopic Arf5 (identified by the HA tag fused to Arf5). Arrowheads indicate paxillin containing plaques at the edge of the cell. (B) Distribution of FAs in U2OS cells expressing Arf5 mutants. The density of paxillin containing plaques at relative distance from the edge of the cell is plotted, 0 being the edge of the cell. 36, 36, 36, and 38 cells were analyzed for control, Arf5‐HA, [T31N]Arf5‐HA, and [I46D]Arf5‐HA. (C) Effect of expressing Arf5 and mutants of Arf5 on number and size of FAs in U2OS cells. The number and size of paxillin‐containing plaques in U2OS cells expressing the indicated Arf5 mutants were determined as described in (Chen and Kroog, 2010) and in “Section 2.” (D) Effect of expression Arf5 and Arf5 mutants on bundles of actin between 5 and 10 µm in length. Bundled actin was quantified using ridge detector in ImageJ. (E) Effect of Arf mutants on bundled actin determined by quantifying peaks in line profiles. The number of peaks in nine line profiles in two ROIs/cell was determined as described in “Section 2.” The mean values from three experiments, each analyzing 10–20 cells/condition, are presented. (F) Localization of [I46D]Arf5‐HA and ASAP1. Representative images are shown. Examples of apparent colocalization of [I46D]Arf5 with ASAP1 at cell edge are indicated with arrows. (G) Colocalization of Arf and Arf mutants with ASAP1. Cells expressing the indicated recombinant Arf were stained for the epitope tag on Arf (HA) and ASAP1. Colocalization was assessed by determining Pearson's coefficients. (H) Immunoblot of cell lysates. Antibodies specific for Arf5 and for the HA epitope were used to detect Arf5. Note the epitope tagged protein has a fusion of a 6 Histidine tandem followed by the HA epitope, runs more slowly than endogenous Arf5. β‐Actin was used as a loading control. FA data were analyzed by two‐way analysis of variance (ANOVA). Other experiments were analyzed by one‐way ANOVA. *p < 0.05; **p < 0.01; ***p < 0.001; ****p < 0.0001.

Arf5 mutants had a greater effect on the actin cytoskeleton than did Arf1 mutants. Arf5‐HA accumulated around the nucleus while [T31N]Arf5‐HA was diffusely distributed through the cell (Figure 5A). [I46D]Arf5‐HA accumulated perinuclearly and at the cell edge with paxillin and actin (inset in Figure 5A). [T31N]Arf5‐HA and [I46D]Arf5‐HA reduced FAs and SFs whereas expression of Arf5‐HA had no effect on FAs or SFs compared to cells transfected with the control (empty) vector (Figure 5C–E). In addition, the residual FAs observed in [T31N]Arf5‐HA and [I46D]Arf5‐HA expressing cells were concentrated in the cell periphery (Figure 5A, B). We examined localization of Arf1‐HA, [I46D]Arf1‐HA, Arf5‐HA, and [I46D]Arf5‐HA relative to ASAP1. Colocalization of Arf1‐HA and Arf5‐HA with ASAP1 was either minimal or did not occur. Because the I46D mutants are resistant to GAP activity of ASAP1 but bind to ASAP1 with the same affinity as does WT Arfs, they might not be readily released from ASAP1 thus accumulate with ASAP1 at the site of ASAP1 action. [I46D]Arf5‐HA colocalized with ASAP1 at the cell edge, with a low but significant Pearson's coefficient that was greater than Arf1‐HA, [I46D]Arf1‐HA, or Arf5‐HA (Figure 5F, G). The combined results support the ideas that (i) Arf5 has a greater role in controlling actin cytoskeleton than does Arf1, (ii) Arf5 might be the physiologic substrate for ASAP1, and (iii) simple control of Arf•GTP levels is not adequate to regulate actin structures, otherwise [I46D]Arf5‐HA (GTP locked) would not have the same effect as [T31N]Arf5‐HA (GDP locked).

### 3.5 Maintenance of SFs and FAs Requires Both Actin Binding and GAP Activity of ASAP1

**FIGURE 6:**
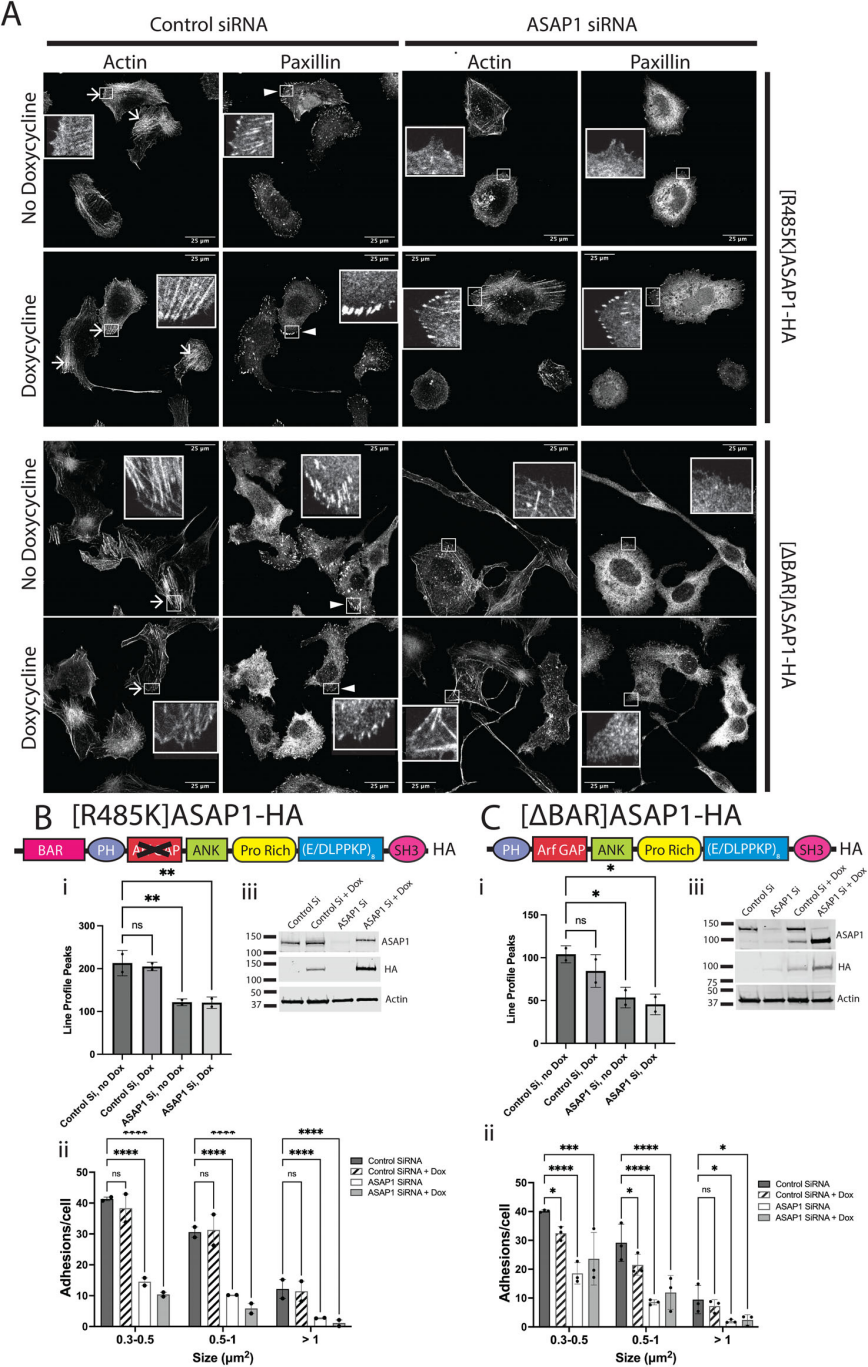
Effect of ASAP1 on actin cytoskeleton and focal adhesions (FAs) requires GAP activity and the BAR domain. Lentivirus was used to integrate cDNA with the open reading frame for the indicated mutants of ASAP1 ([R485K]ASAP1‐HA or [ΔBAR]ASAP1‐HA) under control of a tet‐on promoter. Each cell line was treated with either nontargeting (control) or siRNA targeting the 3′UTR of ASAP1 (ASAP1 SiRNA) for a day, and then treated with either doxycycline (0.5 µg/mL) or vehicle for an addition 2 days before plating on fibronectin coated covers for 6 h, fixing, staining for actin and paxillin, and imaging. (A) Representative images. Examples of stress fibers (SFs) are indicated by arrows, FAs by arrowheads. (B) Effect of reduced ASAP1 and expression of [R485K]ASAP1‐HA on SFs and FAs. (i) Bundled actin, quantified by determining the number of peaks from line profiles, was taken as a measure of SFs. The summary of two experiments, analyzing 10–20 cells/condition/experiment, is shown. (ii) FAs. Summary data from two experiments for [R485K]ASAP1 and three experiments for [ΔBAR]ASAP1 is presented. (iii) Protein levels determined by immunoblotting. Total ASAP1 (includes endogenous and the mutants) was detected with an antibody to ASAP1. The doxycycline‐induced protein was detected with an antibody to the HA epitope tag. β‐actin was used as a loading control. (C) Effect of reduced endogenous ASAP1 and expression of [ΔBAR]ASAP1‐HA on SFs and FAs. Subpanels (i–iii) parallel those in Part B.

We examined the roles of the Arf GAP activity and the BAR domain of ASAP1 for the maintenance of SFs and FAs. Previously we reported that actin binding to the BAR domain was necessary for SFs, but FAs were not examined (Gasilina et al. 2022). GAP activity has been implicated in the maintenance of FAs (Randazzo et al. 2000) but SFs were not examined. Here, using U2OS cells with doxycycline‐inducible ASAP1‐HA, [ΔBAR]ASAP1‐HA, which binds neither actin nor NM2, and [R485K]ASAP1‐HA, which lacks GAP activity, we examined both FAs and SFs. As in the parental U2OS cells, reduced ASAP1 expression resulted in reduced FAs and SFs (Figures 2 and 6). The effects on FAs and SFs were reversed by ectopic expression of WT ASAP1 (Figure 2) but not by [ΔBAR]ASAP1‐HA or [R485K]ASAP1‐HA (Figure 6). Expression of [ΔBAR]ASAP1‐HA also had a small effect reducing FAs and SFs in cells with endogenous ASAP1, possibly resulting from displacing the endogenous protein. In some cells expressing [R485K]ASAP1‐HA, some paxillin‐containing plaques occurred but were not attached to robust SFs (see inset in Figure 6 in panels for ASAP1 siRNA and Dox treatment).

### 3.6 Maintenance of SFs and FAs Require the BAR Domain and GAP Activity of ASAP1 Reside in the Same Polypeptide

**FIGURE 7:**
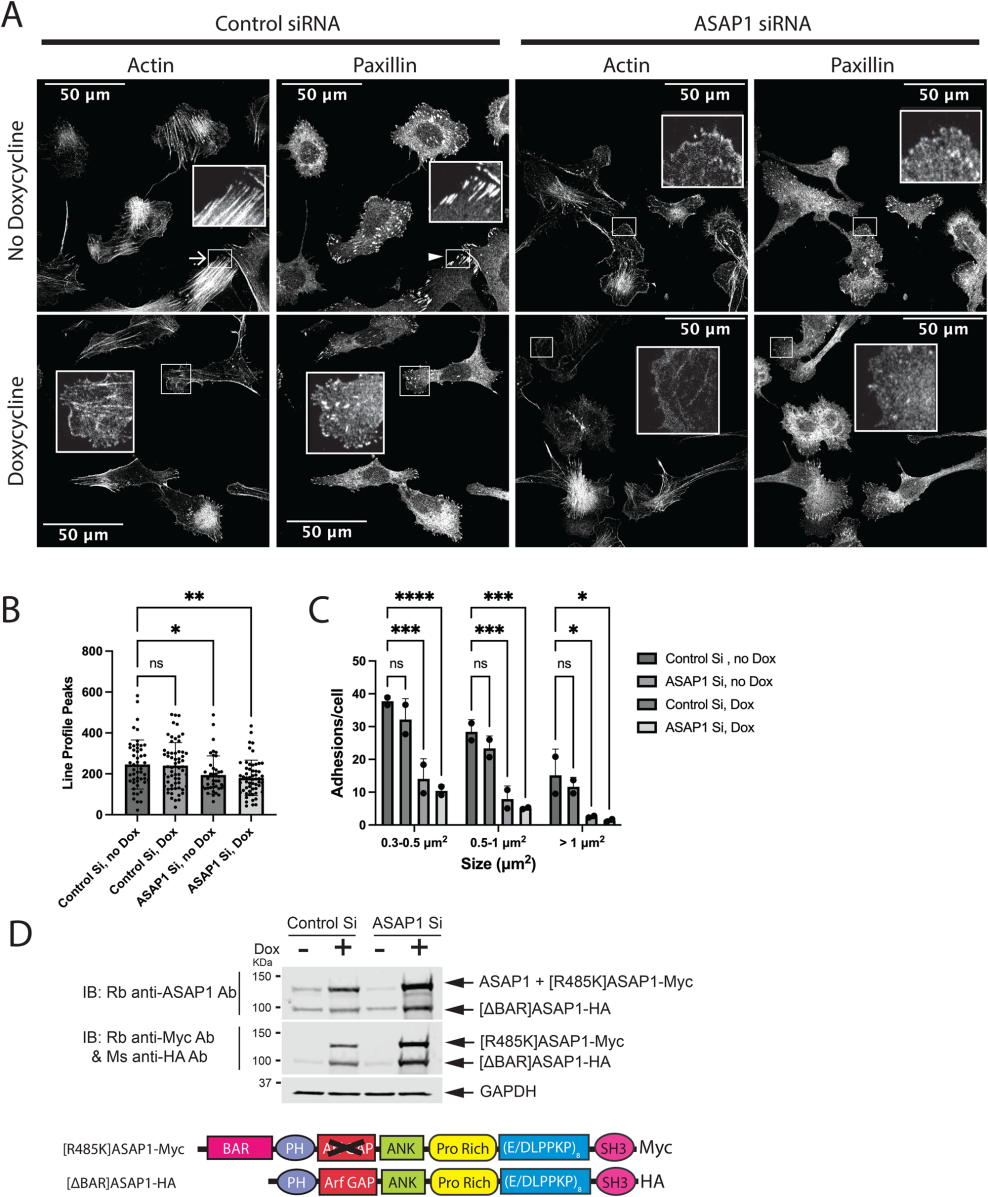
BAR and GAP domains must be in the same polypeptide to support formation of focal adhesions (FAs) and stress fibers (SFs). Lentivirus was used to integrate cDNA with open reading frames for both [ΔBAR]ASAP1‐HA and [R485K]ASAP1‐myc under control of a tet‐on promoter into the chromosome of U2OS cells. Cells were treated with either nontargeting siRNA (control) or siRNA targeting the 3′UTR of ASAP1 (ASAP1 siRNA) for 1 day before addition to vehicle or 0.5 µg/mL doxycycline for 2 additional days before plating on fibronectin‐coated coverslips for 6 h, fixing and staining for actin and paxillin. (A) Representative images. Examples of SFs are indicated with arrows, examples of FAs are indicated with arrowheads. (B) Bundled actin. Quantification from two experiments, with 10–20 cells/condition/experiment of the number of peaks from line profiles was determined as described in “Section 2.” (C) FAs in cells expressing mutant ASAP1. Summarized data from two experiments is shown. (D) Protein levels in U2OS cells. Immunoblot was used to assess the expression of the mutant ASAP1. Proteins were detected with an antibody against a C‐terminal epitope of ASAP1, which would detect both endogenous and ectopically expressed ASAP1 mutants, or antibodies to the epitope tags. GAPDH was used as a loading control. Note that the point mutants express to higher levels compared to the endogenous protein. Also, the expression of the mutants is greater than that achieved when expressing wild‐type ASAP1 (see Figure 3). Furthermore, expression of the ASAP1 proteins from the tet‐on promoter was greater when endogenous ASAP1 was reduced. This phenomenon is being examined in another project. Bundled actin was analyzed by one‐way analysis of variance (ANOVA), FA data by two‐way ANOVA. *p < 0.05; **p < 0.01; ***p < 0.001; ****p < 0.0001.

**FIGURE 8:**
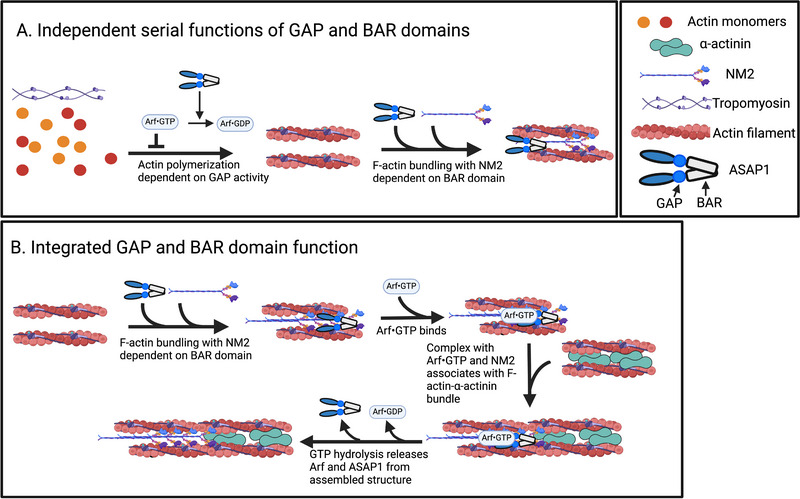
Examples of possible parallel and integrated mechanisms for ASAP1 control of stress fiber (SF) dynamics. (A) Parallel function of the BAR and GAP domains. If reduction of Arf•GTP levels increases actin polymerization, as described for Git (Nishiya et al. 2005), then Arf GAP activity would promote the formation of actin filaments. In parallel, the BAR domain promotes actin bundling necessary for the formation of stress fiber precursors that are subsequently assembled into stress fibers. (B) Integrated activity of the BAR and GAP domains. Based on the findings that GTP binding and hydrolysis are necessary for SFs, that both actin binding and GAP activity are necessary and that the two activities be in the same polypeptide, we propose ASAP1 regulates SFs by a mechanism that integrates the two activities. One possible mechanism is illustrated. In Step 1, ASAP1 bundles actin. In Step 2, Arf•GTP binds to ASAP1. The complex of actin/ASAP1/NM2 then fuses end‐to‐end with another stress fiber precursor containing α‐actinin. Hydrolysis of GTP on Arf results in dissociation of ASAP1 from the complex. Note that this is one possibility of many but might be a reasonable starting hypothesis for understanding the function of ASAP1 in the control of cytoskeleton dynamics. We do not include focal adhesions (FAs) in this model. We speculate that the changes in FAs are secondary to SFs.

The two functions of ASAP1, that is, (i) binding F‐actin and NM2 and (ii) inducing hydrolysis of GTP on Arf, could be independent of each other (illustrated in Figure 8), in which case expressing two proteins, one with a functioning BAR domain and one with GAP activity, is predicted to rescue the loss of endogenous ASAP1. The idea was tested using U2OS cells expressing [ΔBAR]ASAP1‐HA and [R485K]ASAP1‐myc under a tet‐on promoter (Figure 7). As observed in other U2OS‐derived cell lines, the reduction of endogenous ASAP1 expression results in a loss of FAs and SFs. Inducing expression [ΔBAR]ASAP1‐HA and [R485K]ASAP1‐myc together did not reverse the effect of ASAP1 knockdown. There are several possible explanations. There might be a difference in targeting when the protein lacks the BAR domain, although in preliminary studies and previous work, the SH3 and proline‐rich domains were sufficient for targeting. It is also plausible that the function of the two domains is integrated, as described in “Discussion,” below, with one possibility illustrated in Figure 8. Distinguishing among the possibilities is a subject of ongoing work that is outside the scope of the current study.

## 4 Discussion

The dynamic regulation of FAs and SFs is essential for mammalian cells. The structures participate in cell survival, differentiation signaling, proliferation signaling, cell and tissue morphology, and cell migration. The central importance of the structures is reflected in the complex regulation, involving extracellular signals and multiple intracellular signaling pathways, including Ras, Rho, receptor tyrosine kinases, Src family kinases, and focal adhesion kinase (FAK). ASAP1 might be integral to the regulation. It is a target of Src family kinases and FAK. It binds to other proteins that regulate FAs and SFs, including CrkL and p85 PI kinase. It also binds directly to F‐actin and NM2A. Here, we find that ASAP1 colocalizes with paxillin, NM2A, α‐actinin, and actin in FAs, and might colocalize with NM2A and α‐actinin in SFs and structures that might be SF precursors. The BAR domain and GAP activity were both necessary for ASAP1 function in maintaining FAs and SFs. Notable in the images is that ASAP1‐containing puncta partly overlap with both α‐actinin‐containing puncta and NM2A‐containing puncta. The results have led us to hypothesize that ASAP1 might directly mediate the assembly of higher order actin structures in a mechanism where ASAP1 simultaneously binds NM2‐bundled actin and α‐actinin bundled actin. The association is controlled by Arf•GTP and fusion of the two bundles occurs on GTP hydrolysis (Figure 8). Hence, ASAP1 activity is controlled by Arf, rather than Arf being controlled by ASAP1.

Several lines of evidence support the idea that ASAP1 does not function as a canonical GAP in signal transduction but is instead mediating effects potentially under the control of Arf. First, an effector role of ASAP1 has been invoked to explain the similar effect of Arf knockdown and ASAP1 knockdown, although other possible explanations for the result include Arf function in multiple parallel pathways with multiple effectors, some antagonizing the effects of others. A second line of evidence is that the BAR domain binds F‐actin and to NM2, thus potentially having a direct effect on the structures containing these proteins. The codependence of the BAR and GAP domains on the PH domain is additional evidence of a direct functional role of ASAP1 on actin cytoskeleton under the control of Arf. The PH domain of ASAP1 is the Arf binding site in ASAP1. It is necessary for GAP activity (Soubias et al. 2024) and for function of the BAR domain (Chen et al. 2020; Chen et al. 2016; Gasilina et al. 2019; Kam et al. 2000), suggesting an integrated module of the BAR domain (possible effector domain), the PH domain (the Arf binding site), and the GAP domain. Additional results related to the function of ASAP1 is that an Arf mutant that does not load with GTP and an Arf mutant that binds to the GAP but does not hydrolyze GTP have overlapping effects, suggesting an activity linked to the cycle of GTP binding and hydrolysis by Arf. One hypothesis for integrated function is illustrated in Figure 8. In this case, ASAP1 might be thought of as an effector, but not in the canonical sense for GTPases. ASAP1 and Arf do not function through a linear signaling cascade. Instead, ASAP1 mediates an activity, for example, assembly of a bundled actin structure, that relies on both binding Arf•GTP and hydrolyzing the GTP bound to Arf. In the hypothesis, Arf•GTP binding allows ASAP1 to simultaneously bind NM2‐bundled actin and α‐actinin bundled actin, with GTP hydrolysis leading to the fusion of the bundles and dissociation of ASAP1. ASAP1 might attach SFs to nascent adhesions by a similar mechanism. The cycle can mediate multiple assembly events, analogous to the cycle of GTP binding and hydrolysis by EFTu mediating multiple rounds of peptide bond formation (Thompson 1988). We speculate that ASAP1 functions at two points. First, it forms a complex with bundled actin. Second, Arf•GTP promotes the association with a second bundled actin structure, for example, at an FA. Hydrolysis of GTP on Arf releases Arf and ASAP1 from the assembled structure, allowing another cycle of assembly. In this hypothesis, an inability to bind Arf or hydrolyze GTP could trap ASAP1 at the edge of the cell in nascent adhesive structures, for instance, similar to what we have observed here. Mutants of ASAP1 defective either in inducing GTP hydrolysis on Arf or binding to actin would either no replace WT protein and, possibly, might block function of the WT protein; consequently, they would no rescue reduced expression of endogenous ASAP1, as we observed. Furthermore, the term GAP, which implies function to control Arf•GTP levels, might be a misnomer, named for the first biochemical activity observed but not related to its actual function in controlling assembly of higher order actin structures; the misnaming seems fitting given that Arf was named for a pathophysiologic activity used to identify a heterotrimeric G protein, and not for its biological functions, which were discovered years later (Donaldson et al. 1992; Donaldson et al. 1991; Kahn and Gilman 1984; Kahn et al. 1992; Schleifer et al. 1982; Serafini et al. 1991).

Other Arf GAPs might function by similar mechanisms. More than half of Arf GAPs have the structure of an N‐terminal domain‐PH domain‐Arf GAP tandem. The 6 with BAR domains N‐terminal to the PH domain might function with actin and NM2. Analogous to the Arf GAPs with BAR domains that bind to NM2, the AGAPs with a Miro domain N‐terminal of the PH domain, bind to kinesins, motor proteins that function with microtubules. Considering noncanonical relationships between Arf and its GAPs might provide insight into the control of FAs. At least six Arf GAPs are in FAs (ASAP1, ASAP3, ARAP2, Git1, Git2, and AGAP2) (Ha et al. 2008; Hoefen and Berk 2006; Liu et al. 2002; Vitali et al. 2017; Zhao et al. 2000; Zhu et al. 2009). Each could function as an effector controlling a specific activity contributing to regulation of FAs. For instance, ASAP1 controls SFs connected to the FAs while ARAP2 controls integrin recycling (Chen et al. 2014; Chen et al. 2013; Yoon et al. 2006), critical for maintenance of FAs. The mechanism might extend to GAPs outside of the Arf GAPs, for example, GRAF1, GRAF2, and oligophrenin, which have a similar domain architecture as ASAP1 with BAR, PH, GAP, and SH3 domains (Billuart et al. 1998; Doherty et al. 2011; Lundmark et al. 2008; Shibata et al. 2001), and Nadrin and SH3BP1 with BAR and Rho GAP domains (Harada et al. 2000; Parrini et al. 2011).

In our hypothesis, Arf does not function as a switch in the same sense as Ras or heterotrimeric G proteins (Downward 2003; Mosaddeghzadeh and Ahmadian 2021; Takai, Sasaki, and Matozaki 2001; Zebisch et al. 2007). Instead, Arf function is more analogous to EFTu. Ribosomes induce GTP hydrolysis on EFTu to control polypeptide synthesis as part of a proof‐reading mechanism (Maracci and Rodnina 2016; Thompson 1988). Another example is signal recognition particle (SRP) functioning with signal recognition particle receptor (SRPR) (Gasper et al. 2009; Saraogi, Akopian, and Shan 2011, 2014), which controls cotranslational translocation of polypeptides across ER membranes. There are other examples of nucleotidases with functions outside of nucleotide metabolism or signaling, for example, motor proteins, ion transporters, and structural proteins (actin, myosin, and septins). In addition to being relevant to the effects of Arf on actin, the idea that the Arf function depends on the cycle of GTP binding and hydrolysis is consistent with the reported effects of Arf1 and ArfGAP1 and 2 in the assembly of cargo laden coated vesicles (East and Kahn 2011; Nie and Randazzo 2006). A canonical signaling function is not completely precluded; there might be some canonical signaling mediated by Arfs, for example, in phospholipid signaling (Boman et al. 1999; Brown et al. 1995; Honda et al. 1999; Shin and Exton 2001; Van Valkenburgh et al. 2001). Nevertheless, also considering noncanonical signaling might be useful for understanding Arf pathways.

In summary, we have found that the GAP and BAR domains coordinate control of actin SFs and FAs. We speculate that ASAP1 regulates SFs under control of Arf, which consequently affect the dynamics of FAs.

## Conflicts of Interest

The authors declare no conflicts of interest.

## Supporting information



Supporting Information
