# Supplementary material for: Actin Binding to the BAR Domain and Arf GAP Activity of ASAP1 Coordinately Control Actin Stress Fibers and Focal Adhesions
Source: Biol Cell. 2025 Apr 7;117(4):e70005. doi: 10.1111/boc.70005 (PMC11975550; doi:10.1111/boc.70005)
Supplement: Supplementary file 1 — Supporting Information [file BOC-117-e70005-s001.docx]

**
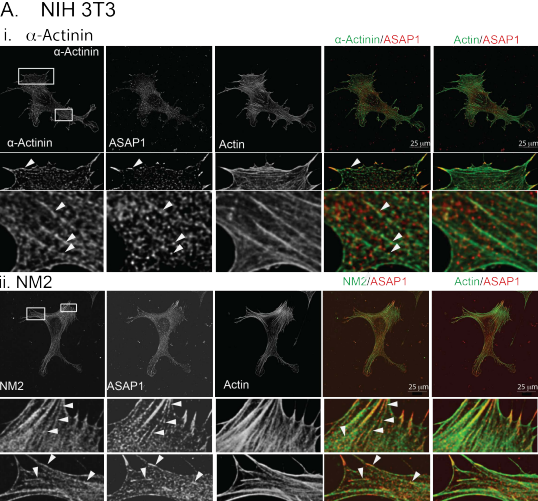
**

**
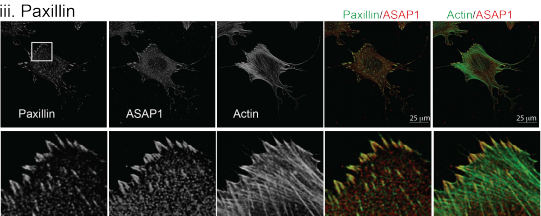
**

**
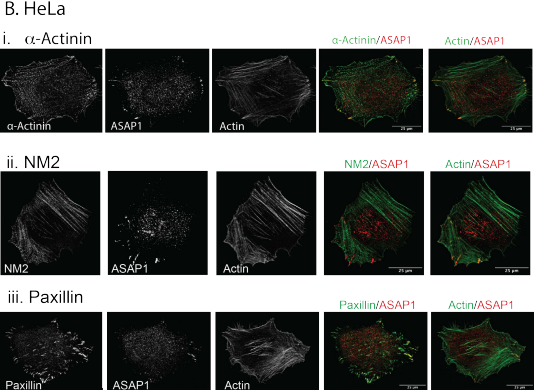
**

**Supplemental Figure S1. Localization of ASAP1 relative to cytoskeleton components. A. NIH 3T3. B. HeLa.** The indicated cells were plated on fibronectin coated coverslips, immunostained for ASAP1 and F-actin, and either NM2A, α-actinin or paxillin and imaged using a Leica TS SP8 confocal laser scanning microscopy with a lightening module. In A, scale bars are 50 μm. In B, scale bars are 10 μm.


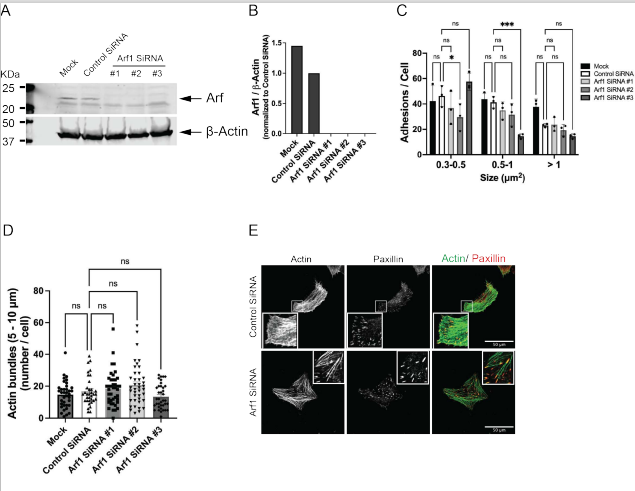


**Supplemental Figure S2. Reduced Arf1 expression does not affect bundled actin and FAs. A. Immunoblot of lysates of cells treated with three different siRNA targeting Arf1.** U2OS cells were treated with either a nontargeting (control) siRNA or one of three different siRNA targeting Arf1, as indicated. After three days, cells were harvested. Aliquots were lysed and immunoblotted. Note that there is a nonspecific band running immediately below the band for Arf1.  **B. Quantification of the Immunoblot.** Signals from fluorescent secondary antibodies were quantified using an Odyssey Imager (LiCOR). β-actin was used as a loading control. **C. Effect of Arf1 knockdown on FAs in U2OS.** U2OS harvested three days after siRNA transfected were plated on fibronectin coated coverslips for 5 hours, fixed, stained for F-actin and paxillin, and imaged. **D. Effect of Arf1 knockdown on SFs in U2OS cells.** Bundled actin fibers between 5 and 10 μm long were determined with RidgeDetector in ImageJ and taken as a measure of SFs. **E. Representative images** of cells with reduced Arf1 expression. FA data were analyzed by two-way ANOVA, actin bundles were analyzed by one way ANOVA. *, p<0.05; ***, p<0.001.

**
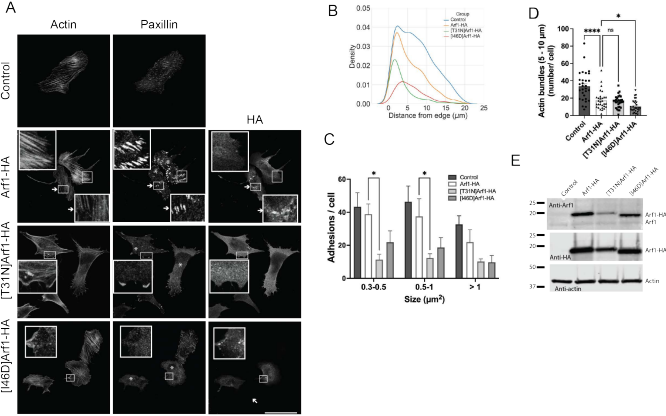
**

**Supplemental Figure S3. Arf1 mutants affect FAs and SFs. A. Representative images of U2OS cells expressing the indicated mutants of Arf1.** Cells were transfected with plasmids for expression of the indicated Arf1 mutant. Control cells were treated with transfection reagent and empty vector. After 3 days, the cells were replated on fibronectin coated coverslips for 6 hrs, fixed and stained for F-actin, paxillin or the ectopic Arf1 (identified by the HA tag fused to the Arf). Asterisks indicate transfected cells. Arrowheads indicate paxillin containing plaques at the edge of the cell. Small arrows point to perinuclear distributed Arf1 or mutant Arf1. **B. Distribution of FAs in U2OS cells expressing Arf1 mutants.** The probability of finding paxillin-containing plaques at relative distance from the edge of the cell is plotted. **C. Effect of expressing Arf1 and mutants of Arf1 on FAs in U2OS cells.** The number and size of paxillin-containing plaques in U2OS cells expressing the indicated Arf1 mutants were determined. **D. Effect of expression Arf1 and Arf1 mutants on bundles of actin between 5 and 10 μm in length.** Bundled actin was quantified using ridge detector in imageJ to analyze the images of cells treated as described in Fig S2A. **E.** Immunoblot of cell lysates. Arf1 and mutants were detected with an Arf1 specific antibody. The epitope tagged proteins are specifically detected with an antibody to the HA tag. Actin is used as a loading control. Note that the ectopically expressed Arf1 with the fusion of a 6 histidine tandem followed by the HA epitope tag is larger than the endogenous Arf1 and, consequently, runs more slowly on the gel. Data for FAs were analyzed by two way ANOVA. Data for actin fibers was analyzed by one way ANOVA. *, p< 0.05; ****, p<0.0001.
